# GPC3 expression in mouse ovarian cancer induces GPC3-specific T cell-mediated immune response through M1 macrophages and suppresses tumor growth

**DOI:** 10.3892/or.2014.3300

**Published:** 2014-07-02

**Authors:** CHENHONG LUO, KIYOSUMI SHIBATA, SHIRO SUZUKI, HIROAKI KAJIYAMA, TAKESHI SENGA, YOSHIHIRO KOYA, MINA DAIMON, MAMORU YAMASHITA, FUMITAKA KIKKAWA

**Affiliations:** 1 Bio-Databases Institute of Reproductive and Developmental Medicine, Nagoya 458-0818, Japan; 2 Department of Obstetrics and Gynecology, Nagoya University School of Medicine, Nagoya 466-8550, Japan; 3 Department of Cancer Biology, Nagoya University School of Medicine, Nagoya 466-8550, Japan; 4 Bell Research Center for Reproductive Health and Cancer, Nagoya 458-0818, Japan

**Keywords:** ovarian cancer, glypican-3, M1 macrophages, heparan sulfate, T cells

## Abstract

Glypican-3 (GPC3) is specifically expressed in ovarian clear cell carcinoma (OCCC), hepatocellular carcinoma (HCC), and melanoma and lung cancer. GPC3 is being explored as a potential candidate for OCCC and HCC immunotherapy. As a tumor-associated antigen, induction of immune response of GPC3 in ovarian cancer remains elusive. We established a GPC3 transgenic mouse ovarian cancer cell line, OV2944-HM-1 (HM-1), and used the intraperitoneal ovarian cancer mouse model to investigate immune response in GPC3-expressing tumor. We found that GPC3 expression in the tumor increased F4/80^+^CD86^+^ macrophage (M1) proportion and caused GPC3-specific CD8^+^ T cell immune responses, and prolonged mouse survival. Our results demonstrated that GPC3 expression induced T cell-mediated immune response in this mouse ovarian cancer model and also provided supportive evidence that GPC3 is an ideal target for ovarian cancer immunotherapy.

## Introduction

Glypican-3 (GPC3), a member of the glypican family of heparan sulfate proteoglycans (HSPGs) which are attached to the cell surface by a glycosylphosphatidylinositol (GPI) anchor (1), is specifically expressed in ovarian clear cell carcinoma (OCCC) (2,3), hepatocellular carcinoma (HCC) (4,5), lung cancer (6) and melanoma (7). GPC3 is responsible for the Simpson-Golabi-Behmel syndrome (SGBS) via the Hedgehog signaling pathway (8). GPC3 is identified to be a tumor-associated antigen and stimulates the growth of HCC cells by increasing autocrine/paracrine canonical Wnt signaling (9). In ovarian and lung cancer, GPC3 inhibits the growth of cancer cells by adding 5-aza-2′-deoxycytidine to restore GPC3 expression in cancer cells (11,12). GPC3 is considered as a candidate target for immunotherapy of OCCC and HCC (13–16). Clinical trials of GPC3-based immunotherapy for HCC are currently being conducted (17,18).

GPC3 is an HSPG. Hence, it is considered to participate in inflammatory response in tumor tissue. Heparan sulfate in syndecans binds to chemokines and cytokines through positively charged domains and thus regulates the activity of chemokines through formation of chemokine gradient (19–23). The interactions modulate inflammatory responses. Cell surface HSPGs also influence MHC class II-restricted antigen presentation (24). GPC3 expression in HCC causes macrophage increase and it is involved in the recruitment of M2 macrophages (25,26). However, it remains elusive in ovarian cancer. Macrophages, a highly heterogeneous cell population, adapt and respond to various microenvironmental signals (27). M1 macrophages phenotypic definition in mouse is iNOS^+^IL-12^+^CD86^+^MHC-II^high^ (28). The M1 macrophages process tumor antigens and present them to lymphocytes. Consequently, T lymphocytes become activated, proliferate, and infiltrate the tumor (29). The M2 macrophages are phenotypically defined as CD206^+^CD163^+^CD36^+^ARG1^+^MHC-II^low^ IL-10^+^IL-4Ra^+^FIZZ1^+^YM1^+^ (28) and can stimulate tumor growth (30); macrophage polarization plays an important role in the tumor growth process.

GPC3 is demonstrated to induce T cell-mediated immune response in HCC (16,31); however, it is unclear in GPC3 expressing ovarian cancer. Therefore, we used mouse GPC3, which shares 95% amino acid sequence identity with human GPC3, established a GPC3 transgenic mouse ovarian cancer cell line, OV2944-HM-1 (HM-1), and used the intraperitoneal ovarian cancer mouse model to analyze immune response in GPC3-expressing mouse ovarian cancer.

## Materials and methods

### Cell line, construction of plasmid, transfection and reagents

The mouse ovarian cancer cell line HM-1 consisting of murine ovarian cancer cells of B6C3F1 origin was provided by Professor Ikuo Konishi (Department of Gynecology and Obstetrics, Kyoto University Graduate School of Medicine, Japan). The cells were cultured in minimum essential medium-α (Sigma-Aldrich, Irvine, UK) supplemented with 10% fetal calf serum (FCS; Gibco^®^ Life Technologies, Grand Island, NY, USA) in a humidified atmosphere of 5% CO_2_ at 37°C. The GPC3-overexpressing HM-1 cells were derived from the HM-1 cell line by stable transfection of a GPC3-containing pcAGGS-IRES-puro vector. The vector and an empty vector were kindly provided by Dr T. Nakatsura (National Cancer Center Hospital East, Chiba, Japan). The vectors were used to transfect HM-1 cells using Lipofectamine (Invitrogen). Clonal selection was performed in a 7 μg/ml puromycin (Sigma-Aldrich, St. Louis, MO, USA)-containing medium. Selected colonies were assayed using western blotting; a puromycin-resistant colony transfected with the empty vector was used as a control. Anti-GPC3 antibody (cat. no. ab66596) was purchased from Abcam Ltd. (Cambridge, UK). Anti-GAPDH (14C10; cat. no. 2118) was purchased from Cell Signaling Technology Inc. (Beverly, MA, USA). Anti-mouse F4/80 labeled with fluorescein isothiocyanate (FITC; cat. no. 122606) or red-algae phycoerythrin (PE; cat. no. 123109), Alexa Fluor 647-labeled anti-mouse CD86 (cat. no. 105019), allophycocyanin (APC)-labeled CD206 (cat. no. 141707), PE-(cat. no. 123109)-labeled anti-mouse interferon-γ (IFN-γ; cat. no. 506507), and FITC-labeled anti-mouse CD8a antibodies (cat. no. 100803) were purchased from BioLegend (San Diego, CA, USA).

### Animals

Six-week-old female B6C3F1 mice were obtained from SLC (Hamamatsu, Japan) and kept in appropriate facilities at the Bell Research Center for Reproductive Health and Cancer. The mice were kept under a 12/12-h light/dark regime in standard cages and handled according to the guidelines of the Institute, with the approval of the Local Animal Care Ethics Committee. The mice were anesthetized by subcutaneous injection of 7.5% chloral hydrate (Wako, Japan).

### In vivo tumorigenicity

Female B6C3F1 mice were used for the assessment of tumor formation. The control and GPC3-expressing HM-1 cells were injected intraperitoneally (1×10^6^ cells in 0.1 ml PBS). The mice were examined each day for tumor ascite accumulation or followed up for mouse survival rates. Approximately 1 week after inoculation with the cancer cells, visible ascites were detected by abdominal swelling and the mice were sacrificed. The peritoneal cavity was opened. Ascitic fluids were collected using a 1-ml syringe.

### Western blot analysis

The control and GPC3-expressing HM-1 cells were cultured in 35-mm dishes overnight. The cells were washed three times with PBS and solubilized in 200 μl RIPA buffer [50 mM Tris-HCl (pH 7.4), 150 mM EDTA, 1% Triton, 1% sodium deoxycholate, and 0.1% SDS]. Western blotting was performed using standard protocols with antibodies against GPC3 and GAPDH.

### Preparation of peritoneal macrophages and isolation of cells from tumor tissues

Mouse peritoneal wash or cancer ascetic fluid was collected and centrifuged. The collected cells were stained for FACS. The peritoneal macrophages were used for the co-culture assay and *in vitro* phagocytic assay. To obtain tumor-infiltrating lymphocytes, tumors were excised and minced on ice. Then, RPMI-1640 containing 20% FCS and 200 U/ml of collagenase from *Clostridium histolyticum* (Worthington Biochemical Corporation, Lakewood, NJ, USA) was added, and suspensions of tumor were incubated for 2 h at 37°C. The suspensions were passed through a sterile 100-μm BD Falcon™nylon mesh (BD Biosciences Labware, Bedford, MA, USA) for debris removal. The cells were treated with 1× BD Pharm Lyse™ (BD Biosciences, San Jose, CA, USA) lysis buffer at room temperature for 5 min and then washed two times with RPMI-1640 (PAA Laboratories GmbH, Pashing, Austria). The cells were stained as described below.

### Isolation of lymph node and spleen cells

Lymph node cells were obtained from inguinal lymph nodes. The collected lymph nodes or spleens were crushed through a 40-mm nylon cell strainer (BD Biosciences Labware, Bedford, MA, USA). Erythrocytes were depleted using the 1× BD Pharm Lyse™ lysis buffer, and the cells were suspended in 10% FCS-containing RPMI-1640 for the antigen stimulation test or stained directly for FACS analysis.

### Immunofluorescence staining and flow cytometry (FCM)

Cells (5×10^5^) were washed with PBS containing 1% bovine serum albumin (BSA; Wako, Japan) and stained with one or two labeled antibodies. Nonspecific FcR binding was blocked by rat serum. At least 10,000 cells were assayed by FCM using BD FACSAria II (BD Biosciences, San Jose, CA, USA) and the data were analyzed using the FlowJo data analysis software package (TreeStar, Ashland, OR, USA). Nonviable cells were visualized by adding 0.5 μl of 7-AAD Viability Staining Solution (BD Biosciences).

### Cell proliferation assay

A water-soluble tetrazolium (WST-8) (Kishida Chemical Co., Ltd., Osaka, Japan)-based colorimetric proliferation assay was performed according to the manufacturer’s instructions. Cells (5×10^4^ cells/well) were plated on 24-well plates. Replication assays were performed at 4, 24, 48, and 72 h.

### Co-culture assay

Peritoneal macrophages (1×10^6^) were seeded in a 35-mm dish, and a Transwell insert (0.4-μm pore; Nunc) containing 5×10^5^ cancer cells was inserted into the dish. The cells were incubated at 37°C and the macrophages were harvested on the indicated days for CD86 analysis by FACS.

### In vitro phagocytic assay

Fluorescent labeling of cells with green fluorescent dye carboxyfluorescein diacetate succinimidyl ester (CFSE) was performed according to the manufacturer’s protocol. CFSE-labeled HM-1 or HM-1GPC3 (1×10^6^) cells were incubated with peritoneal macrophages (5×10^6^); the cells were harvested on day 3 and stained with anti-mouse F4/80-APC prior to flow cytometric analysis.

### Immunofluorescence staining of tumor tissues and inguinal lymph nodes

Mouse intraperitoneal tumors were excised. Frozen sections were fixed with cold acetone for 15 min, blocked with 5% rat serum in PBS, reacted with FITC-labeled anti-mouse F4/80 antibody at 37°C for 30 min. The sections were then washed three times with PBS, incubated with Hoechst 3358 (Invitrogen, Eugene, OR, USA), and mounted with FluoroShield (ImmunoBioScience, Mukilteo, WA, USA). Finally, the slides were examined using an Olympus Fluoview FV1000-D laser-scanning confocal microscope (Olympus Co., Tokyo, Japan).

### Splenocyte stimulation assay

Mouse splenocytes were isolated on day 7 after intraperitoneal injection of cancer cells (1×10^6^ cells). The splenocytes (5×10^6^ cells) were incubated at 37°C for 2 h and stimulated with freeze-thawed HM-1GPC3#1 lysate overnight in the presence of 1× monensin solution (cat. no. 420701; BioLegend). The stimulated splenocytes were harvested, fixed in 2% paraformaldehyde for 10 min at room temperature, washed with 1 ml of 5% BSA in PBS containing 0.5% saponin (Saponin buffer; Wako Chemicals Inc., Richmond VA, USA) and stained with anti-mouse PE-labeled IFN-γ, APC-labeled CD3, and FITC-labeled CD8a in saponin buffer on ice for 30 min. The cells were analyzed using a flow cytometer after washing with PBS containing 1% BSA. The same experiment was performed as described without adding monensin and then the cultured medium was collected for IFN-γ determination with ELISA kit.

### Cytokine detection

To detect tumor necrotic factor-α (TNF-α), interleukin-12 (IL-12), or IFN-γ levels in ascitic fluids or medium, ELISA kits were used according to the manufacturer’s instructions (BioLegend).

### TUNEL and immunohistochemical staining

Frozen sections were fixed in 4% PFA for 10 min. After washing in PBS, the sections were digested in proteinase K (40 mg/ml; Sigma Chemical, St Louis, MO, USA) for 15 min. Cell death was assessed using the ApopTag^®^ Peroxidase In Situ Apoptosis Detection Kit (Millipore Corporation, Billerica, MA, USA), according to the manufacturer’s recommendations. In brief, the slides were incubated in a humidified chamber for 1 h at 37°C with a reaction mixture containing TdT and biotinylated deoxyuridine triphosphate to label the exposed 3′-hydroxyl ends of nicked single-strand DNA. The antibody complexes were detected using the ABC streptavidin-horseradish peroxidase Kit and developed using DAB (Vector Laboratories Inc., Burlingame, CA, USA).

### Statistical analysis

The significance of changes in cell replication, cytokine secretion, and macrophage percentages on individual days was determined using Student’s t-test. Cell proliferation was analyzed by one-way ANOVA. Survival rates were estimated by the Kaplan-Meier method, and significance was determined by the log-rank test. Cell replication O.D., cytokine concentrations in wash solutions and ascitic fluids, and CD86^+^/F4/80^+^ macrophage percentages are expressed as means ± SD. Statistical analysis was performed using SPSS 1.1J Software^®^ (SPSS Inc., Chicago, IL, USA). Differences with P<0.05 were considered statistically significant.

## Results

### GPC3 expression increases macrophage recruitment into tumor masses

**Figure 1:**
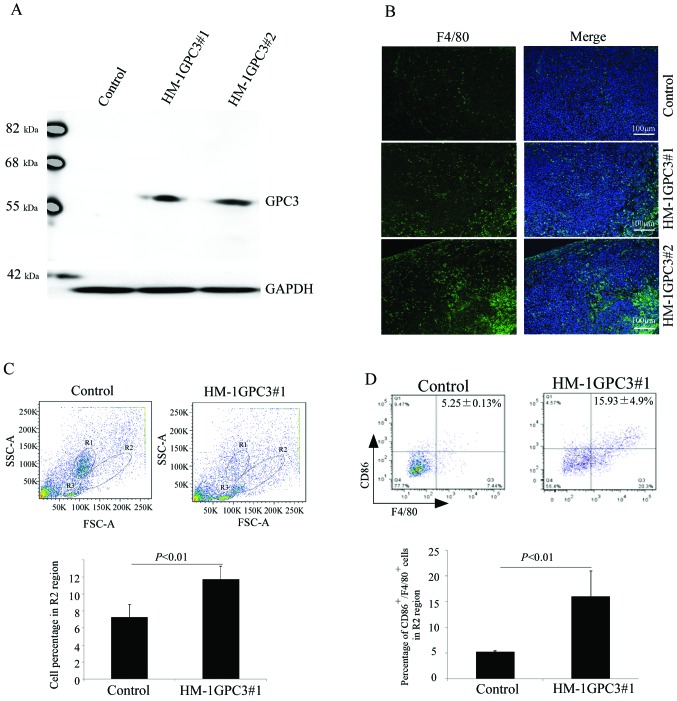
GPC3 transgenic HM-1 cell generation and GPC3 overexpression induces M1 macrophage infiltration. Two colonies of cells stably expressing GPC3 (HM-1GPC3#1 and HM-1GPC3#2) were selected, and GPC3 expression was confirmed using western blotting with an anti-GPC3 antibody (A). Tumor-tissue sections collected 8 days after inoculation with cancer cells were stained with an anti-F4/80 antibody (light spot). Cell nuclei were counterstained with Hoechst 3358. Images were captured using a confocal microscope at ×200 magnification (B). FCM of infiltrating macrophages in control and HM-1GPC3#1 tumors 8 days after inoculation with 1×10^6^ cancer cells; cytograms show morphological parameters of cell size (FSC) and cell granularity (SSC) of dissociated cells (top panel in C). In dot plots on ungated samples, lines were drawn around different cell populations; lymphocytes (R3), monocytes and macrophages (R2), and granulocytes (R1). The percentage of cells in R2 regions is presented in the graph (bottom in C). Tumor-infiltrating macrophages were analyzed with anti-CD86 and F4/80 antibodies using FCM (top panel in D). The graph presented in the bottom section in D contains combined reproducible data (n=3). Results are mean value ± SD. (n=3).

We established a GPC3 transgenic HM-1 cell line as described in the Materials and methods section. We confirmed GPC3 expression in two colonies (HM-1GPC3#1 and HM-1GPC3#2) using western blotting with an anti-GPC3 antibody (Fig. 1A). Therefore, we used the intraperitoneal GPC3-expressing ovarian cancer mouse model to examine macrophage distribution in tumor with a mouse F4/80 monoclonal antibody. HM-1GPC3#1 and HM-1GPC3#2 tumors had higher F4/80-positive cells compared with control tumors (Fig. 1B). Moreover, macrophages were examined by FCM on day 8 after cancer cell inoculation (Fig. 1C). Upon tumor dissociation, since leukocytes can be distinguished from malignant cells on the basis of their size and morphology using FACS analysis (FSC-A) (SSC-A), we identified regions of lymphocytes (R3), monocytes and macrophages (R2), and granulocytes (R1) (Fig. 1C, top). Results indicated that GPC3 expression induced an 11.7% increase in macrophage and monocyte recruitment compared with the control (~7.3%) regarding the total of number of cells collected from dissociated tumors (Fig. 1C, bottom graph). In this study, we used CD86 and F4/80 as markers for identifying M1 macrophages in the R2 region. The percentage of infiltrating CD86^+^/F4/80^+^ cells in the GPC3-expressing tumor was approximately 15.93% in the region of monocytes and macrophages (Fig. 1D), whereas it was only 5% in the control tumors. Fig. 1D (bottom graph) presents the percentage of CD86^+^/F4/80^+^ cells, and results indicated a significant difference (^*^P<0.05). Data were obtained from three independent experiments.

### GPC3 expression increases the number of CD86^+^/F4/80^+^ macrophages in ascitic fluids and enhances the secretion of IL-12 and TNF-α cytokines

**Figure 2:**
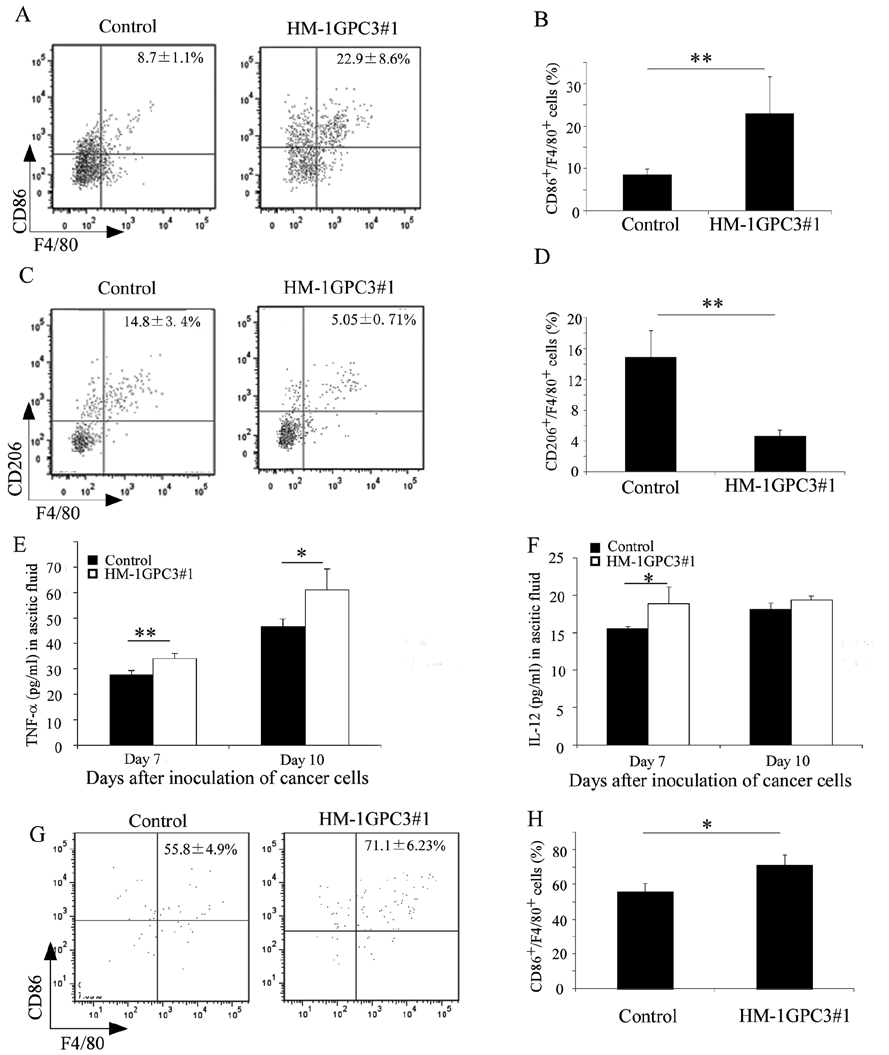
GPC3 expression increases the percentage of CD86^+^/F4/80^+^ cells and facilitates TNF-α and IL-12 secretion in ascitic fluids. Macrophages collected in washes on day 3 after inoculation were analyzed with anti-CD86 and F4/80 antibodies (A) or anti-CD206 and F4/80 antibodies (C) by FACS. Graphs are representative of results shown in Fig. B and D. TNF-α (E) and IL-12 (F) levels in ascitic fluids were determined by ELISA. Inguinal lymph node cells were analyzed with anti-CD86 and F4/80 antibodies on day 4 after inoculation with cancer cells by FCM (G and H). Error bars for B, D–F and H represent SD (n=3). Data are from three independent experiments. ^*^P<0.05, ^**^P<0.01.

We collected peritoneal washes or ascitic fluids on days 3, 7 and 10 after inoculation with cancer cells for the determination of TNF-α and IL-12 cytokines, which are secreted by M1 macrophages, and for the analysis of macrophage properties. Our data revealed CD86^+^/F4/80^+^ (M1) and CD206^+^/F4/80^+^ (M2) cells in peritoneal washes and ascitic fluids 3 and 7 days after inoculation with cancer cells. On day 3 after inoculation, the CD86^+^/F4/80^+^ cells amounted to 22.9% of the total cell number in the regions of monocytes and macrophages in the GPC3-expressing group. However, the CD86^+^/F4/80^+^ cells constituted only 8.7% in similar regions in the control group (Fig. 2A and B). On day 7 after the inoculation with cancer cells, no significant difference was observed in the percentage of CD86^+^/F4/80^+^ cells (P>0.05) between the GPC3-expressing and control groups (data not shown). The percentage of CD206^+^/F4/80^+^ cells was 5.05% in the GPC3-expressing group and 14.7% in the control group on day 3 after inoculation with cancer cells (Fig. 2C and D). We also determined that the secretion levels of TNF-α (Fig. 2E) and IL-12 (Fig. 2F) were significantly higher in the ascitic fluids of the GPC3-expressing HM-1 cell-infected mice than in the control HM-1 cell-injected mice on day 7 after inoculation with cancer cells. However, there was no significant difference in the secretion levels of IL-12 on day 10 after inoculation with cancer cells. We also examined the percentage of CD86^+^/F4/80^+^ cells in inguinal lymph nodes on day 4 after inoculation with cancer cells. Our results indicate that the CD86^+^/F4/80^+^ cells were significantly more abundant in the lymph nodes of the GPC3-expressing group than in the control group (Fig. 2G and H). The percentage of CD86^+^/F4/80^+^ cells in inguinal lymph nodes was 71.1% in the GPC3-expressing group and 55.8% in the control group.

### GPC3 expression increases CD86 expression in peritoneal macrophages and increases HM-1 cell susceptibility to phagocytosis by mouse peritoneal macrophages in vitro

**Figure 3:**
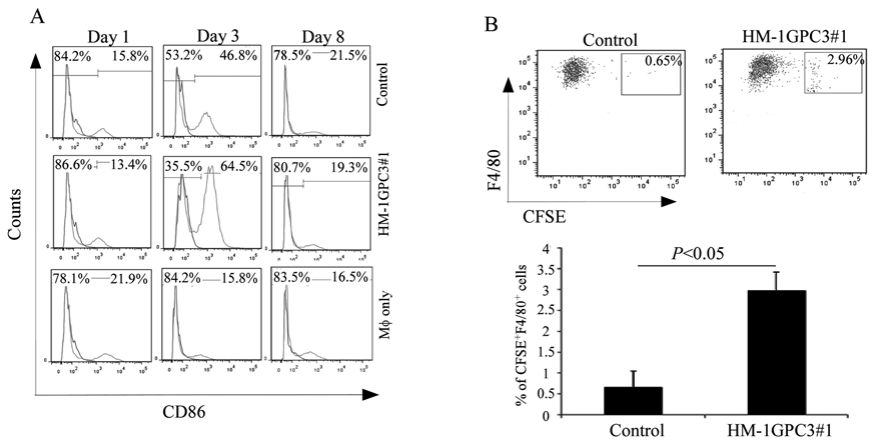
GPC3 induces CD86 expression in mouse peritoneal macrophages and facilitates phagocytosis of HM-1 cells by peritoneal macrophages *in vitro*. Macrophages were co-cultured with mouse ovarian cancer cells and analyzed for CD86 expression at indicated time points. Numbers represent the percentage of CD86-expressing macrophages. The dark line represents isotype control staining; the light line indicates positive CD86 staining (A). CFSE-labeled control or HM-1GPC3#1 cells (1×10^6^) were incubated with mouse peritoneal macrophages (5×10^6^) at 37°C; cultures were harvested 3 days later and phagocytosis was determined by FCM. Data are presented from three independent experiments. Results are mean value ± SD (B).

An increased number of CD86^+^ macrophages were present in the GPC3-expressing tumor. Therefore, we investigated the effect of GPC3 on CD86 expression *in vitro*. The collected mouse peritoneal macrophages were co-cultured with the control HM-1 cells and GPC3-expressing HM-1 cells in Transwell inserts without direct cell-cell contact. We collected co-cultured macrophages for CD86 expression analysis by FCM. The results showed that, on day 3 of co-culture, GPC3 expression increased the proportion of CD86-expressing peritoneal macrophages to approximately 64.5%. Co-culture with the control HM-1 cells induced the number of CD86-expressing macrophages to approximately 45%. No changes were observed in macrophage-only cultures (Fig. 3A). Moreover, to determine whether expression of mouse GPC3 on HM-1 cells facilitated phagocytosis by mouse peritoneal macrophages, we performed *in vitro* phagocytic assay. Mouse peritoneal macrophages were markedly more effective in engulfing HM-1GPC3#1 cells than in engulfing the control HM-1 cells (Fig. 3B).

### GPC3 expression increases CD8^+^ T cell infiltration, cellular immune induction and the number of apoptotic cancer cells in the tumor mass

**Figure 4:**
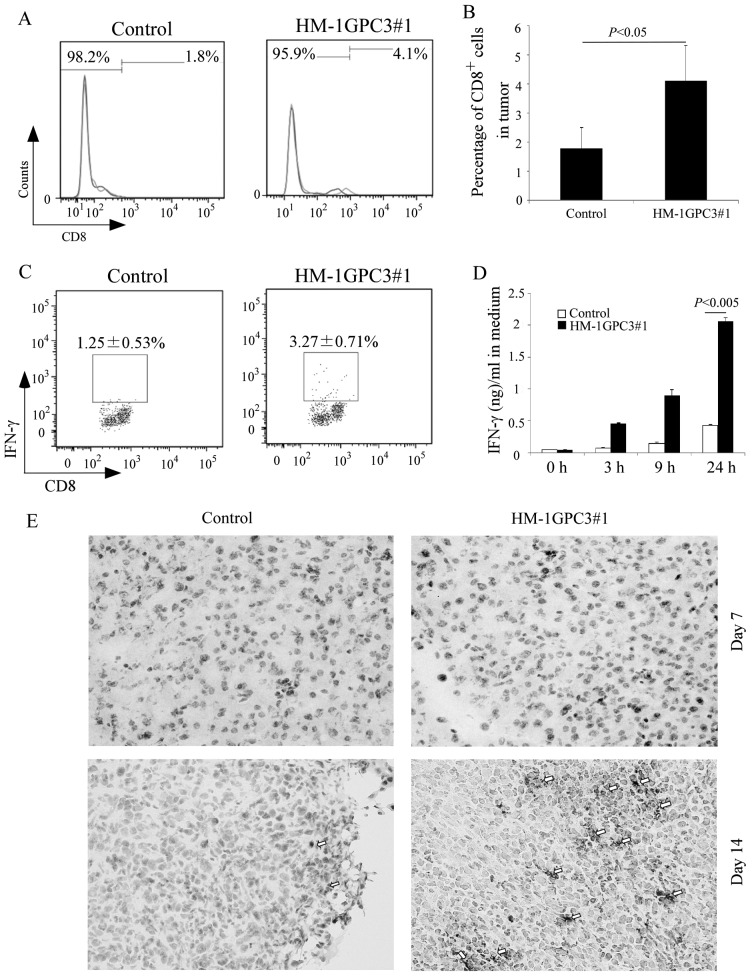
GPC3 overexpression induces CD8^+^ cell infiltration and enhances cellular immune response to GPC3. The percentage of tumor-infiltrating CD8^+^ cells is shown in A and B. Results are representative of three independent experiments. Splenocytes were isolated on day 7 after inoculation with cancer cells and were stimulated with GPC3-expressing HM-1 cell lysate for 6 h in the presence of monensin. Then, they were immunostained with anti-IFN-γ and CD8 antibodies for FACS (C). Using ELISA kits, IFN-γ levels were measured in the culture medium of splenocytes at indicated times after stimulation with GPC3-expressing HM-1 cell lysate. Results are representative of three independent experiments (D). TUNEL assay of apoptotic cells in tumor tissue. TUNEL labeling of apoptotic cells in tumor-tissue samples obtained from mice 7 and 14 days after inoculation with cancer cells in peritoneal cavities (E). Error bars for B and D, represent SD.

To perform their immune function, T lymphocytes are activated by M1 macrophages (29). Therefore, we analyzed CD8^+^ T cell infiltration and tumor apoptosis. Peritoneal tumor masses were removed 14 days after inoculation with cancer cells and disassociated. Disassociated tumor cells were stained with anti-CD8 FITC-conjugated antibody and analyzed by FACS. The FACS analysis showed that CD8^+^ T cells constituted 4.1% of the total lymphocytes in the GPC3-expressing tumor and 1.51% in the tumor formed by injection of the control HM-1 cells (Fig. 4A and B). To evaluate cellular responses against GPC3, we also stimulated splenocytes from mice inoculated with the control HM-1 cells and HM-1GPC3#1 cells by adding freeze-thawed HM-1GPC3#1 cell lysate. Then, we examined IFN-γ production kinetics. We found that more IFN-γ accumulated in the medium of splenocytes from the HM-1GPC3#1-inoculated mouse group than from the control group (Fig. 4D). Therefore, we used the flow cytometric method to evaluate the contribution of CD8 subset of T lymphocytes in the spleen to the immune response to the GPC3-expressing HM-1 cells. We measured the production of intracellular IFN-γ in this subset after brief antigenic stimulation. In splenocytes from the mouse spleen inoculated with the GPC3-expressing HM-1 cells, 3.27% of the CD8-subset cells of T lymphocytes were IFN-γ positive. In controls, 1.25% of the CD8-subset cells were IFN-γ positive (Fig. 4C). We also measured the incidence of cancer cell apoptosis by conducting the TUNEL assay using tumor tissue extracted on days 7 and 14 after inoculation with cancer cells. The TUNEL assay of HM-1GPC3#1 tumor sections showed more apoptotic nuclei (arrow heads) than tumor sections obtained from mice injected with the control HM-1 cells and the increase in the number of apoptotic nuclei was time-dependent (Fig. 4E).

### GPC3 expression prolongs mouse survival and decreases formation of ascitic fluids in vivo

**Figure 5:**
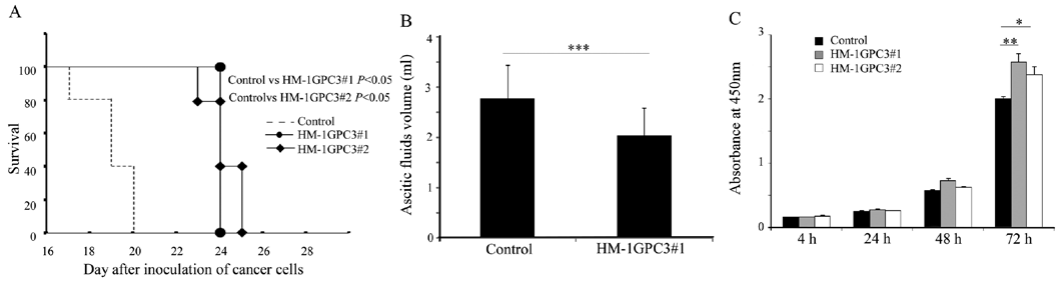
GPC3 overexpression inhibits tumor growth. The Kaplan-Meier survival curve for each inoculation group represents the percentage of surviving animals at the indicated time points (1×10^6^ cells of each tumor cell line were used to inoculate the peritoneal cavity. n=5/each group) (A). On day 8 after inoculation with tumor cells, mice (10/each group) were sacrificed, peritoneal cavities were opened, and ascitic fluids were collected and analyzed (B). Cancer cells were seeded in a 24-well plate in triplicate and cultured at 37°C. Cell growth was determined at 4, 24, 48, and 72 h using the WST-8 method. Significance was assessed by one-way ANOVA (C). ^**^P<0.01, ^*^P<0.05. Error bars for B and C, represent SD.

The two colonies of GPC3-expressing HM-1 cells had significantly higher proliferation rates than the control cells at 72 h after seeding *in vitro* (HM-1 vs. HM-1GPC3#1 or HM-1GPC3#2; P<0.01 or 0.05) (Fig. 5C). GPC3 expression significantly improved survival time (HM-1 vs. HM-1GPC3#1 or HM-1GPC3#2; P<0.05). All of the control HM-1 cell-injected mice died by day 20 after inoculation. However, the mice inoculated with the GPC3-expressing HM-1 cells died by day 24 or 25 after inoculation (Fig. 5A). The growth rates of the HM-1GPC3#1 and HM-1GPC3#2 cells were similar both *in vivo* and *in vitro*. We compared the formation of ascitic fluid of the HM-1GPC3#1 and the control HM-1 cells *in vivo*. The formation of ascitic fluid was less pronounced in the HM-1GPC3#1-injected mice (Fig. 5B). Thus, GPC3 significantly suppressed tumor growth.

## Discussion

GPC3 expression on a cell membrane stimulates recruitment of macrophages into HCC tissues (10). Therefore, we used this mouse ovarian cancer model to analyze the effect of GPC3 on ovarian cancer immune microenvironment. According to our analysis, GPC3 expression significantly increased the proportion of M1 macrophages in tumor mass and ascitic fluids. Macrophages are a highly heterogeneous cell population. M1 macrophages process tumor antigens and present them to lymphocytes. M2 macrophages stimulate tumor growth (30). Hence, our results suggest that GPC3 expression increases the number of M1 macrophages in ascitic fluids, especially at an early time. Furthermore, we examined the cytokines TNF-α and IL-12 in ascitic fluids, which are secreted by M1 macrophages (32). We found the two cytokines were higher in ascitic fluids of GPC3 expression cancer than the control especially at an early time; it was similar to M1 macrophages in ascitic fluids. These results further suggest that GPC3 expression increases the proportion of M1 macrophages. Moreover, previous studies demonstrated that the combination of tumor immunotherapy with IL-12 and TNF-α may be more effective to mouse melanoma (33), and heparan sulfate causes the release of TNF-α and IL-12, and induces cytotoxic capability in peritoneal macrophages (34). Our results also revealed GPC3 expression HM-1 cell phagocytosis by intraperitoneal macrophages *in vitro*. These studies provide evidence to support that GPC3 as an HSPG may suppress mouse ovarian cancer growth through increasing macrophage phagocytosis and induction of IL-12 and TNF-α secretions.

CD86 is a protein expressed on antigen-presenting cells that provides costimulatory signals necessary for T cell activation and survival (35). In this study, we used CD86 as a marker to determine M1 macrophages. In addition, as a result of F4/80^+^CD86^+^ macrophage increase in GPC3-expressing mouse ovarian cancer, we analyzed the effect of GPC3 on CD86 expression in mouse intraperitoneal macrophages. We obtained a result that GPC3 on mouse ovarian cancer cells induces CD86 expression on mouse intraperitoneal macrophages. Soluble heparan sulfate delivers signals to macrophages and upregulates MHC-II and CD86, markedly increasing the ability of the macrophages to modify immune responses (34). The increase in CD86 expression by virtue of GPC3 on mouse peritoneal macrophages may enhance the antigen-presenting ability of macrophages.

M1 macrophages are antigen-presenting cells that process tumor antigens and present them to lymphocytes after migration into the draining lymph nodes (29). According to our results, M1 macrophages in GPC3 expressing cancer ascitic fluids increase on day 3 after inoculation with cancer cells. We also confirmed that M1 macrophages increase significantly in inguinal lymph node on day 4 after inoculation with cancer cells and F4/80^+^ cells migrate into T cell zone of the inguinal lymph nodes at the same time (data not shown). Thus, the time- and space-dependent distribution of F4/80^+^CD86^+^ cells suggests that the increase in the abundance of F4/80^+^CD86^+^ cells in the inguinal lymph nodes might be associated with the migration of F4/80^+^CD86^+^ cells from the peritoneal cavity. These results provide further evidence to support that GPC3 expression recruits M1 macrophages and enhances antigen presentation ability to lymphocytes.

Enhancement of antigen presenting capability of macrophage can activates helper T cells and then helper T cells help CD8-T cell responses (36). Our results demonstrated that GPC3-expression increases M1 macrophages in tumor mass, ascitic fluids and inguinal lymph node. Thus, we consider that T cell-mediated immune responses to GPC3 can be induced. In order to confirm this hypothesis, we analyzed tumor infiltrating CD8^+^T cells and found infiltrating CD8^+^T cells increase in GPC3-expressing tumor. In addition, we also confirmed that HM-1GPC3 lysate stimulates splenocytes from the mice inoculated with HM-1GPC3 to secrete IFN-γ specifically. These results demonstrated that GPC3 expression in mouse ovarian cancer is able to induce GPC3-specific T cell-mediated immune response. Effecter T cells kill targets by apoptosis (37). Consequently, we found GPC3 expression increases apoptotic cells in tumor masses. Collectively, we suggest that GPC3 expression recruits M1 macrophages to engulf cancer cells and enhances antigen presentation ability to lymphocytes, and then induces GPC3-specific T cell-mediated immune response to suppress GPC3-expressing tumor growth.

Finally, in order to confirm that this immune response suppresses GPC3-expressing tumor growth, we used the intraperitoneal ovarian cancer mouse model to investigate the effect of GPC3 on mouse survival rate. We found that GPC3 expression decreased ascitic fluid production and significantly prolonged mouse survival. Contrarily, GPC3 expression stimulated cancer cell growth *in vitro*.

In conclusion, the present study demonstrates that GPC3 expression induces T cell-mediated immune response in this mouse ovarian cancer model and also provides supportive evidence that GPC3 is an ideal target for ovarian cancer immunotherapy.

## Abbreviations

GPC3: glypican-3
HSPG: heparan sulfate proteoglycan
OCCC: ovarian clear cell carcinoma
HCC: hepatocellular carcinoma
TNF-α: tumor necrotic factor-α
IL-12: interleukin-12
IFN-γ: interferon-γ
